# HLA alleles and haplotypes in Sudanese population and their relationship with Mediterraneans

**DOI:** 10.1038/s41598-023-40173-x

**Published:** 2023-09-27

**Authors:** Fabio Suarez-Trujillo, Sayda El-Safi, Ignacio Juarez, José Palacio-Gruber, Alejandro Sanchez-Orta, José Manuel Martin-Villa, Antonio Arnaiz-Villena

**Affiliations:** 1 Department of Immunology, School of Medicine, University Complutense, Pabellón 5, Planta 4. Avda. Complutense S/N, 28040 Madrid, Spain; 2 Instituto de Investigación Sanitaria Gregorio Marañón, Madrid, Spain; 3 Ibn Sina Specialized Hospital, Khartoum, Sudan

**Keywords:** Genetics, Molecular biology

## Abstract

The contribution of migrated people from once green Sahara (about 10,000–6000 years bc) towards Mediterranean area had probably a double effect: both genetic and cultural connections have been described between Western Europe and North Africa. Sudanese populations from different ethnicities have been studied for HLA-A, -B, -DRB1 and -DQB1 antigens by a standard microlymphotoxicity method. Results found show that Nubians are genetically related with African Sub-Saharan populations and distant from other Sudanese tribes, who are closer to Mediterranean populations than to Sub-Saharan ones. This is concordant with other authors and meta-analysis data. Our present work is, to our knowledge, the first and only one HLA research that studies Sudanese people according to different Sudan ethnic groups: samples were collected before Sudan partition between North and South. A prehistoric genetic and peoples exchange between Africa and the Mediterranean basin may be observed and is supported with the results obtained in this Sudanese HLA study. However, demic diffusion model of agriculture and other anthropological traits from Middle East to West Europe/Maghreb do not exist: a more detailed Sahel and North African countries ancient and recent admixture studies are also being carried out which may clearer explain pastoralists/agriculture innovations origins in Eurafrican Mediterranean and Atlantic façade.

## Introduction

**Figure 1:**
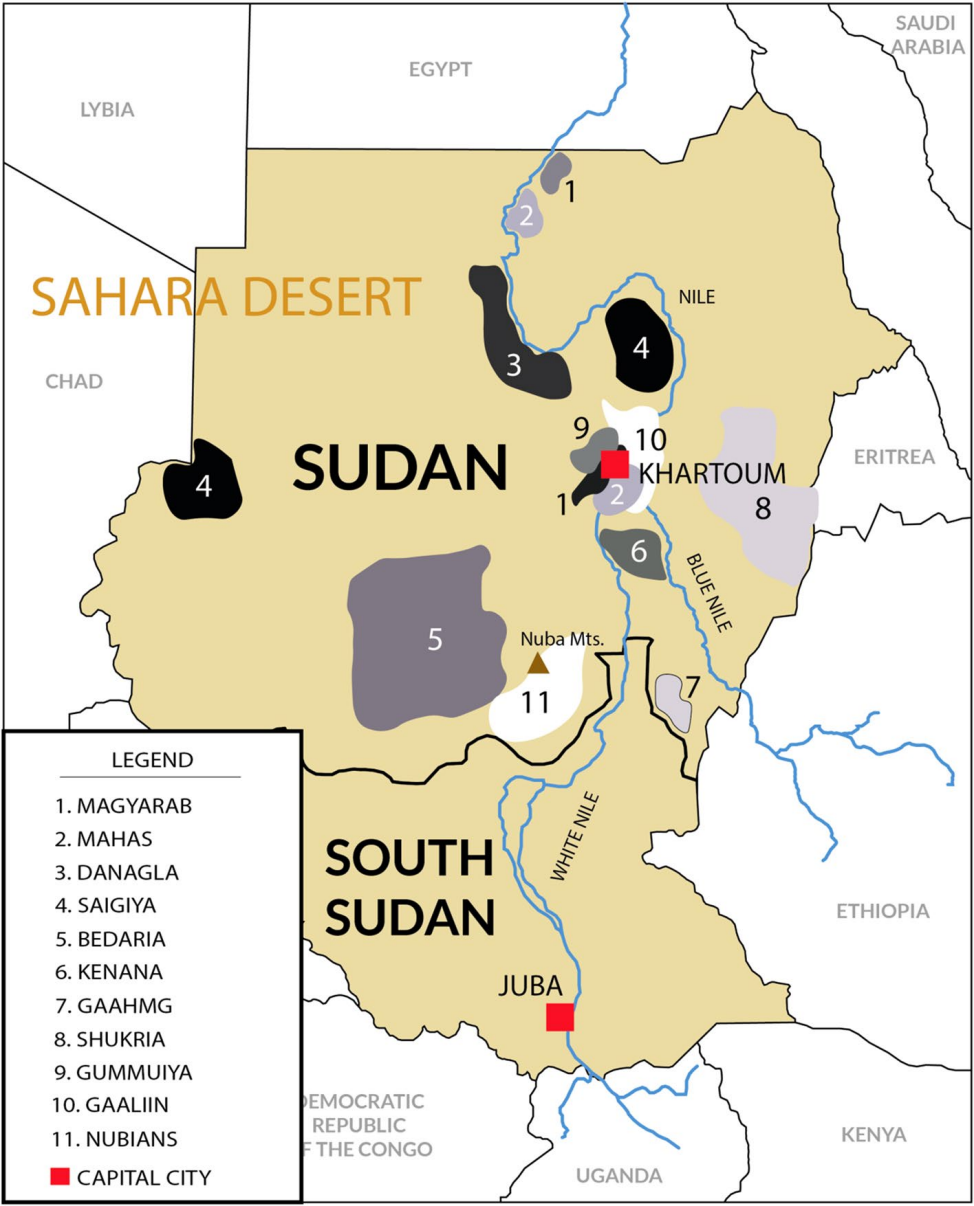
Sudan and South Sudan map (split in 2011). Sudanese tribes included in this study are grey-scale remarked in the figure. Rufaa and Kawahla tribes (not shown in figure) also participated in this study and are widespread all across Central, North and South parts of Sudan. Number of individuals of each ethnicity included in present work were: Magyarab (n = 8), Mahas (n = 7), Danagla (n = 7), Saigiya (n = 9), Bedaria (n = 8), Kenana (n = 10), Gaahmg (n = 6), Shukria (n = 8), Gummuiya (n = 7), Gaaliin (n = 8), Rufaa (n = 11), and Kawahla (n = 9). Nubians (n = 101) HLA genetic data were taken from 12th Histocompatibility International Workshop. For more information see^3^ and www.joshuaproject.net. This figure has been made by our own work based on a map (https://www.rawpixel.com/image/8905866/sudan) and performed with Adobe Illustrator 2020 software (24.3 version; https://www.adobe.com/es/products/illustrator.html).

Sudan means “land of black people” in Arabic language. It became independent from United Kingdom in 1956. It has been involved in several civil wars mainly with North (Muslim) part of Sudan against South (Christian). Two different countries arose in 2011: Sudan at North, mostly of Muslim religion and capital at Khartoum, and South Sudan, mostly of Christian and ancestral autochthonous religions and capital at Juba^1,2^. Samples studied in this work are from 13 different ethnicities of Sudan and were collected from Khartoum before Sudan split that was and still is being a big immigration city; it is placed at the convergence of Blue and White Nile River. Main local tribes or ethnic groups which participated in this study are defined by languages and depicted in Fig. 1 (for more details of Sudan ethnic groups see^3^ and www.joshuaproject.net). Also, South Sudan was somewhat neglected and stayed backwards between the Anglo (UK)-Egyptian mandate during 1899 and 1956. UK and Egypt centred benefits in North part of Sudan, particularly in Khartoum area [1.2]. Ethnicities admixture makes it difficult to assess individual genetic ancestry, although it was established mainly by language and place of origin. The term “Nubian” was used at the time of this study sample collection for African people with some Ethiopian phenotype characters close to Ethiopians or not, but coming from Nuba Mountains, which have blurred limits with South Sudan (Fig. 1).

Sahara desertification became more intense by 10–6000 years ago. Saharan people underwent then a strong migratory pressure and started to emigrate. In the context of a drying Sahara the desert settled populations there began to disperse in all directions, including North Africa and the Mediterranean basin. This gave rise to a mixture of people and genes and influenced the Ancient Classic Mediterranean cultures creating a cultural circle that includes Mediterranean populations and Guanches or Canary Islands First Inhabitants^4,5^. The findings of the same types of Lineal Megalithic or more recent scripts and megalithic structures in the Sahara and throughout the Mediterranean Basin and the Eurafrican Atlantic coast, including Canary Islands, support a common Ancient Megalithic culture that probably was born in the Humid Sahara period before 6000 bc^5^.

In the present study we aim: (1) to compare a mixed sample of different Sudanese ethnicities with other surrounding populations; (2) to clarify their origin according to HLA genetic background and to ascertain their relationships with Mediterranean and Sub-Saharan populations. HLA genes are the transplantation genes in humans and, for strictly biomedical purposes, they are useful compared to other genes for studying human populations and ethnic groups origins because of the HLA very high polymorphism and correlation of HLA allele frequencies follows a geographical gradient^4^; (3) to ascertain genetic differences found between Sudanese Nubians from Nuba Mountains and Sudanese from other Sudan ethnicities (Fig. 1).

## Material and methods

### Populations samples

Ninety-eight healthy unrelated individuals from all Sudan territory have been included in this study belonging to different ethnicities (depicted in Fig. 1): Magyarab (n = 8), Mahas (n = 7), Danagla (n = 7), Saigiya (n = 9), Bedaria (n = 8), Kenana (n = 10), Gaahmg (n = 6), Shukria (n = 8), Gummuiya (n = 7), Gaaliin (n = 8), Rufaa (n = 11), and Kawahla (n = 9). Nubians (n = 101) HLA genetic data were taken from 12th Histocompatibility International Workshop^6^. Blood samples were collected at Ibn Sinna Hospital, in Khartoum State, and used for HLA typing and phylogenetic calculations. Other world populations were considered for genetic comparisons and are detailed in Table 1. The study was approved by Complutense University Ethical Committee (EC-0117.AK) and all subjects included in the study were adult unrelated blood donors who signed an informed consent to participate in it.

**Table 1:** Populations used for the present work.

Population	N	Reference
Sudanese	98	Present study
Lebanese—KZ^1^	93	^6^
Moroccans	98	^7^
Berbers (Souss)	98	^8^
Moroccan Jews	94	^9^
Portuguese	236	^10^
Spaniards	176	^11^
Spanish Basques	80	^11^
French	179	^12^
Algerians	102	^13^
Sardinians	91	^12^
Italians	284	^12^
Ashkenazi Jews	80	^14^
Non-Ashkenazi Jews	80	^14^
Cretans	135	^15^
Macedonians	172	^16^
Attika Greeks	85	^6^
Cypriot Greeks	101	^6^
Aegean Greeks	98	^6^
Romanians	99	^6^
Armenians	97	^6^
Iranians	100	^17^
Turks	89	^18^
Egyptians (Siwa)	101	^6^
Nubians	101	^6^
Oromo	83	^6^
Amhara	98	^6^
Fulani	38	^6^
Rimaibe	39	^6^
Mossi	42	^6^

*N* Number of individuals analysed for each population; ^1^*KZ* Kafar Zubian, a Shia Muslim village.

### HLA typing, DNA sequencing and statistics

Generic HLA class I (A and B) and class II (DRB1 and DQB1) typing was done by two-step microlymphotoxicity technique on T or B lymphocytes^19^ by using the 10th and 11^th^ International Histocompatibility Workshop and local reagents. This technique was used because it was necessary to study all Sudan available ethnicities and HLA low resolution was also available and it is sufficient to accurately distinguish or relate populations^4,11^.

Two types of analyses were done to compare HLA frequencies of Sudan with those of Mediterranean and other populations: (1) with low-resolution DRB1 and DQB1 data; and (2) with low-resolution DRB1 data. This latter analysis was performed in order to compare Nubians from Sudan and other Sudanese ethnicities in a proper way by using the same level of HLA allelism distinction, who have been typed/transformed for HLA-DRB1 generic but lack HLA-DQB1 typing.

Statistical analysis was performed with Arlequin v3.0 software^20^. In summary, this program calculates the linkage disequilibrium between two alleles at two different loci. Their level of significance (p) for 2 × 2 comparisons and also their relative linkage disequilibrium were calculated as previously described^21^. Computer program calculated the most frequent HLA extended haplotypes that were further analysed as follows; the most frequent complete extended haplotypes were tentatively deduced from: (1) the 4 HLA loci haplotype frequencies were obtained by software^21,22^; (2) the previously described haplotypes in other populations were also taken into account^21^; and (3) haplotypes if they appeared in two or more individuals and the alternative haplotype was well defined were also studied^21,22^. In order to compare phenotype and haplotype HLA frequencies with other populations, the reference tables of the 11th and 12th International HLA Workshop were used^6,12^ (Table 1). Phylogenetic trees (dendrograms) were constructed with HLA-DRB1 frequencies by using the Neighbour-Joining (NJ) method^23^ and genetic distances between populations (DA)^24^, by using DISPAN software which contains the programs GNKDST and TREEVIEW^25,26^. Also, correspondence analysis in three dimensions and its bidimensional representation was carried out using Vista v5.05 computer program^27^. Correspondence analysis consists of a geometric technique that may be used for displaying a global view of the relationships among populations according to HLA (or other) haplotypic (or allelic) frequencies. This methodology is based on the genetic distances (DA) variance among populations (similar to the classical principal components methodology) and also a statistical visualization of these differences.

### Ethical approval

This study was conducted according to the Declaration of Helsinki by the Ethics and has been approved by Complutense University Ethical Commitee (EC-0117.AK).

### Consent to participate

All subjects included in the study were adult unrelated blood donors who signed an informed consent to participate in it.

## Results

### Characteristic Sudanese HLA allele frequencies

The expected and observed genotype frequency values for HLA-A, -B, -DRB1 and -DQB1 loci do not significantly differ, and the population sample is in Hardy–Weinberg equilibrium. Table 2 shows the HLA allele frequencies found in our Sudanese analysed sample.

**Table 2:** HLA-A, -B, -DRB1 and -DQB1 allele frequencies found in Sudanese ethnicities sample studied in present work (n = 98).

Allele	%
HLA-A*	
A*02	32.65
A*30	12.24
A*31	10.2
A*03	9.18
A*68	7.15
A*24	6.63
A*01	5.63
A*25	4.08
A*23	3.57
A*32	3.57
A*11	2.04
A*33	1.53
A*29	0.51
A*34	0.51
A*69	0.51
HLA-B*	
B*51	21.43
B*15	8.67
B*49	8.67
B*52	8.17
B*41	6.12
B*57	6.12
B*14	5.63
B*13	3.57
B*38	3.57
B*45	3.57
B*07	3.57
B*44	3.06
B*18	2.04
B*35	2.04
B*50	2.04
B*08	2.04
B*37	1.53
B*42	1.53
B*53	1.53
B*58	1.53
B*55	1.02
B*67	1.02
B*27	0.51
B*39	0.51
B*73	0.51
HLA-DRB1*	
DRB1*13	25.51
DRB1*11	13.27
DRB1*15	13.27
DRB1*03	12.76
DRB1*07	8.67
DRB1*08	8.67
DRB1*04	6.63
DRB1*16	4.08
DRB1*14	3.06
DRB1*10	2.04
DRB1*01	1.53
DRB1*09	0.51
HLA-DQB1*	
DQB1*06	41.84
DQB1*03	27.55
DQB1*02	20.92
DQB1*05	7.65
DQB1*04	2.04

**Figure 2:**
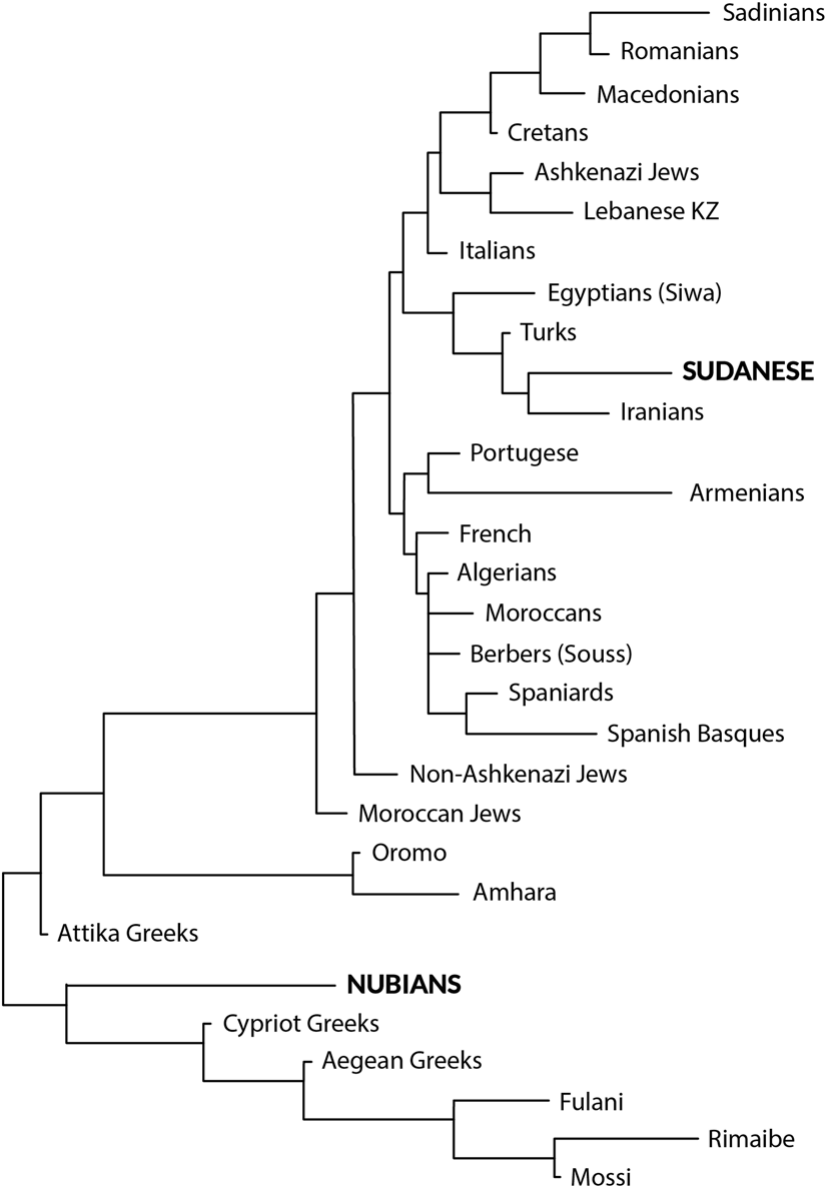
HLA-DRB1 dendrogram performed with Neighbour-Joining method showing genetic relatedness between Sudanese, Nubians, and other Mediterranean and Sub-Saharan populations. Genetic distances between populations were calculated with GNKDST software using low-resolution HLA-DRB1 frequencies. Bootstrap test showed values of 100 in all nodes after 1000 replicates. Note that Greek HLA relatedness with Sahel populations was detected by two independent different groups^4,5,16, 28^ and it is also supported by other autosomal genetic markers for African and Greeks specifically (3120 + 1 G → A cystic fibrosis marker)^29,30^. HLA-DRB1 allele frequencies is used because of the best discrimination between populations among HLA loci (more or less relatedness) and the fact that almost all the populations in data bases are HLA-DRB1 typed^31^.

**Figure 3:**
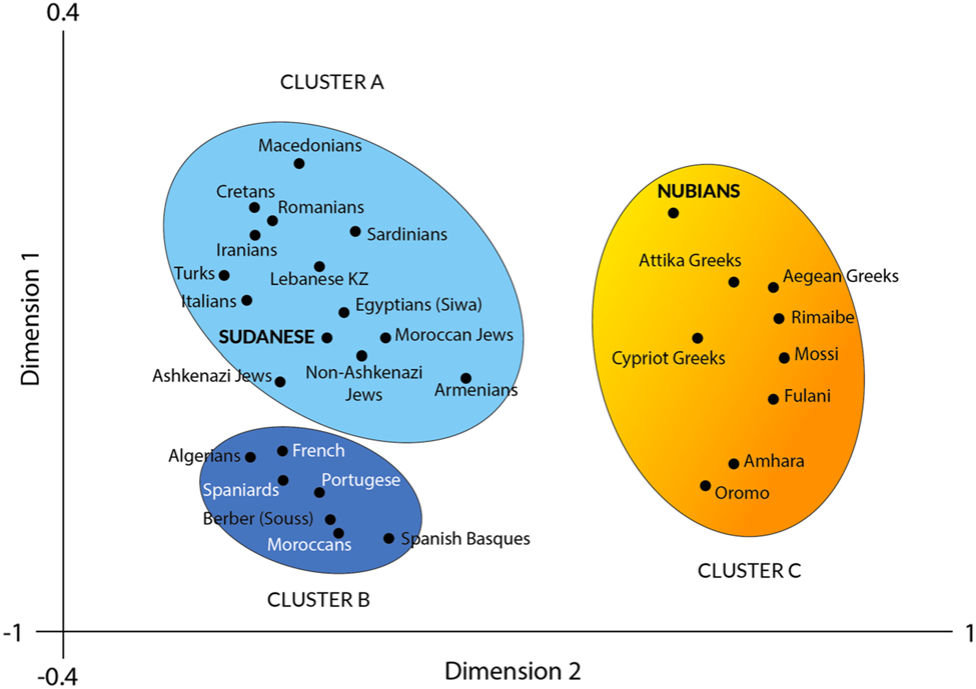
Correspondence analysis performed with HLA-DRB1 allele frequencies of Sudanese sample studied in present work together with those of other Mediterranean and African populations included (see Table 1). Three different clusters are obtained from the analysis: for simplicity, cluster A (light blue), cluster B (dark blue) and cluster C (yellow/orange). Mixed Sudanese population analysed in this study appears together with other Mediterraneans like Turks, Iranians, Egyptians, Italians or Jews in the eastern-Mediterraneans cluster (cluster A, light blue). However, Sub-Saharan Sahelians (Fulani, Rimaibe, Mossi) and Nubians cluster together with Greeks and other African Sub-Saharan populations (cluster C, yellow/orange), as previously obtained by two independent groups^4,5,16,28^. Correspondence analysis supports the results previously obtained in the genetic distances analysis (DA) (Table 3) and NJ dendrogram (see Fig. 2). Italians have been chosen from North Italy^6^, a population that usually cluster with northern Balkan Peninsula or Central European groups.

Figure 2 depicts a low-resolution class II (DRB1) neighbour-joining (NJ) tree constructed form genetic distances between populations (DA, Table 3). Its topology shows how compared populations cluster in two main branches: in general, western (both North African and Europeans) and other Mediterraneans group together respectively and tend to converge in the same node; in the other branch Greeks and Sub-Saharans tend to cluster together with Nubians, Fulani, Rimaibe and Mossi (as described before in^5,16,28^ and with other genetic markers^29,30^. Our Sudanese studied group is placed especially close to most western Mediterraneans: North African (Algerians and Moroccans) and Iberians (Table 3). Genetic distance values (Table 3) give quantitative genetic distances and relatedness between populations and show that Turks are the closest to Sudanese population, followed by Egyptian-Bedouins and Iranians. Also, Mediterranean populations like Algerians, Moroccans, Spaniards and Italians are genetically close to Sudanese population studied in present work. Finally, Greeks and Sub-Saharans cluster together and behave as outgroups. In the same way, the correspondence analysis performed show again how Sudanese are placed within the eastern Mediterranean group, which is also related to the western one (Fig. 3). On the other hand, Nubians cluster together with Greeks and other black Sub-Saharan populations included in this work. Genetic distances between Nubians and our Sudanese mixed sample are similar to those among other Sub-Saharans (Oromo and Amhara) and Sudanese (Tables 3 and 4). These results in Greeks have been previously confirmed by different independent research groups and genes (see “Discussion” section).

**Table 3:** Genetic distances (DA) between studied Sudanese population and other world populations included in this work (HLA-DRB1).

Population	DA (× 10^2^)
Turks	5.91
Egyptians-Siwa	5.93
Iranians	7.44
Algerians	8.42
Moroccans	8.64
Spaniards	8.75
Italians	9.52
Macedonians	10.38
Portugese	10.52
French	10.98
Berbers-Souss	11.6
Cretans	13.28
Romanians	13.30
Non-Ashkenazi Jews	13.41
Ashkenazi Jews	13.57
Moroccan Jews	14.02
Spanish Basques	15.16
Lebanese-KZ	19.71
Sadinians	19.74
Armenians	25.19
Attika Greeks	28.78
Oromo	30.93
Nubians	31.16
Amhara	36.89
Cypriot Greeks	42.71
Aegean Greeks	44.43
Fulani	48.40
Mossi	50.51
Rimaibe	54.79

**Table 4:** Genetic distances (DA) between studied Nubian population from 12th international histocompatibility workshop (n = 101, see^6^) and other world populations included in this work (HLA-DRB1), see Table 1.

Population	DA (× 10)
Greeks (Aegean)	14.84
Greeks (Cyprus)	16.13
Greeks (Attika)	16.61
Rimaibe	19.35
Mossi	23.81
Fulani	33.09
Amhara	20.01
Oromo	20.06
Moroccan Jews	20.15
Macedonians	20.44
Egyptians (Siwa)	20.52
Cretans	23.54
Italians	23.98
Non-Ashkenazi Jews	24.59
Turks	27.67
Portuguese	28.48
French	29.39
Moroccans	29.67
Algerians	30.47
Lebanese (KZ)	31.08
Sudanese	31.16
Romanians	32.52
Berbers (Souss)	33.09
Iranians	33.12
Armenians	34.27
Askenazi Jews	35.32
Sardinians	35.52
Spaniards	40.87
Spanish-Basques	46.44

To complete the genetic relationship study of our studied Sudanese from different ethnicities with other reported populations, a second set of analyses were carried out by using low-resolution HLA-DRB1 data. The constructed NJ tree (Fig. 2) and calculated genetic distances (Tables 3 and 4) show a very similar relatedness of Sudanese population in both types of analyses. Interestingly, Nubians, have a genetic distance value with respect to our Sudanese mixed sample as far as Greeks and Sub-Saharans (Table 3). In fact, when Correspondence analysis with generic HLA-DRB1 data is performed, Nubians are closer to sub-Saharans than any other compared population including our Sudanese mixed-ethnicities studied group (Fig. 3 and Table 4). Table 4 also shows that Nubians are more related to Sub-Saharans and also to Greeks, as it has been demonstrated by us and other researchers^4,5,16^.

### HLA-A, -B, -DR and -DQ linkage disequilibria

The present study reports not only HLA class I (A and B) and class II (DRB1 and DQB1) allele frequencies but also two-loci and extended haplotypes for the first time in a mixed sample of different Sudanese ethnicities, allowing their comparison with previously reported results in other populations.

The most frequent HLA extended four-loci haplotypes (HLA-A-B-DRB1-DQB1) (Table 5) are marked by the high frequency of some of the two-loci haplotypes described above. A*30 and A*31 alleles (low resolution) are particularly frequent in Mediterraneans and also in our Sudanese sample (Table 2); the same occurs with B*49 allele. The most frequent HLA extended haplotype found in our Sudanese sample is A*02-B*51-DRB1*13-DQB1*06 (4.59%) and is identified as a Mediterranean/European haplotype; it is also present in Spaniards (1.0%)^12^. Also, this B-DRB1-DQB1 combination has an Iberian-Mediterranean background because is present with similar frequency in Portuguese, Italians, French, and African Americans from the US^12^. The second most frequent haplotype found in present work (A*02-B*49-DRB1*03-DQB1*02, 2.56%) has been found to be specific of the Sudanese sample studied, although its partial B-DRB1-DQB1 haplotype can be found in Italians^12^. Other Sudanese-specific HLA extended haplotypes with frequencies above 1% have been found in our Sudanese sample: A*02-B*51-DRB1*08-DQB1*03 (1.54%), A*03-B*52-DRB1*11-DQB1*03 (1.54%), and A*01-B*51-DRB1*13-DQB1*03 (1.02%). All other most frequent haplotypes are also shared with Mediterraneans (Table 5).

**Table 5:** Most frequent HLA-A-B-DRB1-DQB1 extended haplotypes in our Sudanese population (n = 98).

Haplotype	%	Origin^28^
A*02-B*51-DRB1*13-DQB1*06	4.59	Mediterranean/European
A*02-B*49-DRB1*03-DQB1*02	**2.56**	**Specific**
A*02-B*51-DRB1*08-DQB1*03	**1.54**	**Specific**
A*03-B*15-DRB1*13-DQB1*06	1.54	European
A*03-B*52-DRB1*11-DQB1*03	**1.54**	**Specific**
A*02-B*50-DRB1*07-DQB1*02	1.02	Mediterranean/South Asian
A*02-B*51-DRB1*13-DQB1*03	1.02	Mediterranean/European
A*02-B*15-DRB1*13-DQB1*05	1.02	European
A*01-B*57-DRB1*15-DQB1*06	1.02	Mediterranean/South Asian
A*01-B*51-DRB1*13-DQB1*03	**1.02**	**Specific**

Significant values are in bold.

## Discussion

### Sudanese HLA Mediterranean background

Our present study shows that Sudanese population is related to Mediterraneans like Turks (who are genetically Mediterraneans except because of an Asian “elite” imposed language^18^), Egyptians, Iranians, Algerians, Italians and Iberians. This is concordant with other North African, Middle East and Iberian HLA compilation study^31^ by using another methodology (high-resolution HLA typing). On the other hand, Greeks were found initially close to Sahel populations (Rimaibe, Mossi, Oromo, Amhara); it disconcerted us, and an allele-by-allele comparison was carried out between Greeks and Sub-Saharans to finally show that they both share some HLA alleles in common, confirming our phylogenetic results^5,16^. Typing of these populations has been done by many participant laboratories in the 12th Histocompatibility International Workshop under the direction of Julia Bodmer (Oxford)^5,6^ and computer calculations were made by us. Also, another autosomal marker (3120 + 1 G → A cystic fibrosis marker) was found in Africans and also only in Greeks out of all other European populations tested^29,30^. It was written by Aeschylus that African peoples (Danaids) emigrated northwards firstly to Egypt, where they were rejected and then fled to Peloponnesus^32^. This may give us a sort of historical reference for these genetic results that have been independently confirmed by other groups and other genetic markers^5,16,28–30^.

### North African migrations and contacts with the Mediterranean Basin

Sahara Desert climate has undergone sharp variations over time, ranging from wet to dry over the past hundreds of thousands of years^33^. This variability is due to a 41,000-year cycle in which the axis of the Earth changes between 22° and 24.5°^33^. In fact, the Columbia Shuttle detected a humid (traces of big rivers and lakes) and green Sahara Desert under the dunes^34^. Currently it is in a dry period, but the Sahara is expected to turn green again in about 15,000 years. Due to these desertification-greening cycles, it is possible that very different human populations inhabited in the last humid stage. The Tassili N’Ajjer National Park and Ahaggar Mountains in Algeria show a record of rock art that proves human settlement in the Sahara^33^. However, when the desertification began around 10,000 bc, the populations that inhabited the Sahara emigrated to other more habitable northern and other areas.

It is well established that North Africans and southern Europeans are genetically related, and this may be due to a long lasting circum-Mediterranean cultural and genetic flow particularly during the last glacial peak^4,35–39^, being a secluded population between European North Ices and Desert. On the basis of our present day genetic and linguistic studies, we have postulated that many people coming from what is nowadays the Sahara Desert started to move towards East, West, North, and also South, being an important part of the primitive people stock of Sumerians, Egyptians, Guanches (Canary Islands), Iberians, Etruscan, Minoans, Anatolians (currently named Turks on only linguistic bases because they show a clear Mediterranean HLA profile)^4^, Kurds, and other islanders or northern Mediterraneans^4,5,18,36–39^. In the present work, HLA genetic background of Sudanese people from different ethnicities is studied, and results obtained inpart confirm these North African-Mediterranean peoples (genes) exchange.

Finally, there are common traits in Mediterranean, Iberian and North African cultures Circle, which may be conjointly named the Saharo-Canarian Circle^40^. Traces of incised lineal writing have recently been found in human sites in the central Sahara (Ti-m Missaou, Algeria) in what would be the first discovery of “megalithic lineal writing” in continental Africa^35^. This finding supports the theory that incised lineal writing was originated in fertile Sahara populations, which moved to the North when desertification began, carrying this type of lineal writing to Canary Islands (Guanche culture), the Mediterranean Basin and Europe. It is probable that these populations of the green Sahara moved towards the Mediterranean and the Canary Islands, originating common cultural and genetic traits among all the descendant populations (Guanches, Egyptians, Minoans, Etruscans, Iberians and Basques among others^38,39^). The present study and other ones^4,5,30,31,34^, support this theory of movements from central Africa to other regions; the appearance of Mediterranean alleles and semi-haplotypes (like HLA-DRB1*03:05, *03:07, *04:11, *04:20, *11:10 or *13:10; results not shown) in different African populations endorse bi-directional population movements from the Sahara to the Mediterranean and South Europe in prehistoric and more recent times. In some cases, Lineal Megalithic Scripts and later classical letters (or syllables) are found together; it is the case of naviform scripts that are found mixed with Iberian-Tartessian semi-syllabary signs in the same context both in Iberian Peninsula and in Canary Islands. An evolutionary pathway of writing may be observed from primitive Lineal Megalithic Scripts to other more recent and complex alphabets or syllabaries like those found in Sitovo^41^, Gradeshnitsa^42^ or Iberian scripts widespread in the Iberian Peninsula, Canary Islands and continental North Africa^43^. Also, other common archaeological elements have been found in Europe and Africa (Canary Islands), including Western Sahara territory^44,45^. The findings pointed out in this study in relation to Sudanese HLA profile support this emigrational theory from the Sahara (and Sub-Saharan areas) to the Mediterranean Basin.

However, other authors with different techniques and genetic markers (SNPs) put forward that admixture with Arabs in recent times has occurred in Sudan and that this area has been subjected to significant population movements in ancient African /Eurasian history^46^. Also, East Sahelians admixture with North Africans is confirmed with other studies^47^, and Fulani nomads, Tuaregs and other Arab speaking Sahelians show admixture with North Africans like most of Sahelian groups do^48^.

## Data Availability

All genotypic data included in present work is held in Allele Frequencies Net Database (http://www.allelefrequencies.net) with population number 3765 and population name “Sudan Khartoum HLA”.
